# Supplementary material for: Fatty acid synthesis suppresses dietary polyunsaturated fatty acid use
Source: Nat Commun. 2024 Jan 2;15:45. doi: 10.1038/s41467-023-44364-y (PMC10762034; doi:10.1038/s41467-023-44364-y)
Supplement: Supplementary file 1 — Supplementary info [file 41467_2023_44364_MOESM1_ESM.pdf]

Inventory of Supporting Information

**Content**	**Page**
Medical history of index patient	2
siRNA list	4
qPCR primer list	4
Taqman assay list	5
Supplemental tables	6
Supplemental Figures	9-24
Supplemental references	25

Supporting information

Medical history of index patient

The boy, the first of two children of non-consanguineous healthy parents from Kongo and Angola (Fig. 1A), presented with moderate delay in speech development, autistic behavior and macrocephaly. He was born after an uneventfully pregnancy at term with birth weight of 3790g (+0.80 z) and occipitofrontal head circumference (OFC) of 35 cm (+0.04 z); birth length was not available. Neonatal period and infancy were unremarkable; all milestones of motor development have been reached in time. However, language development was delayed. The index patient started speaking at 2 years of age and spoke only one- to two-word sentences at the age of 5 years, whereas his receptive language was substantially better. His behavior was described as reclusive and autistic-like. At the last examination at 5 years and 10 months of age, we saw a friendly tall and mildly obese boy with a length of 127 cm (+1.94 z), a weight of 34.6 kg (+2.77 z), a BMI of 21.5 (+1.94 z) and an OFC of 56 cm (+2.52 z). Blood analysis revealed normal thyroid and liver parameters, but slightly increased creatine kinase (207 U/l, n < 148 U/l). Extensive metabolic work up gave normal results. Ultrasound of abdominal organs was normal except for a hyperechogenic signal of liver tissue suggesting mild hepatic steatosis. Brain MRI was inconspicuous. X-ray of the knee revealed genu varum and a hyperostosis of the medial tibia corticalis. Chromosome analysis showed normal male karyotype, CGH-array and fraX-testing were inconspicuous.

To elucidate the cause of developmental delay and behavior problems a trio whole-exome analysis was performed without identification of applicable pathogenic variants in known disease genes. However, a *de novo* missense variant in *FASN* (c.6529C>T, p.Arg2177Cys) was discovered (see also Table S1).

siRNA list

Name	Origin	Kind	Ordered size	Cat#
si*Scr*	Dharmacon	Control pool	20 nmol	D-001206-13-20
si*GPAM*	Dharmacon	SMARTpool	5 nmol	L-009946-00-0005
si*AGPAT1*	Dharmacon	SMARTpool	5 nmol	L-003810-00-0005
si*AGPAT2*	Dharmacon	SMARTpool	5 nmol	L-003811-00-0005
si*LPIN1*	Dharmacon	SMARTpool	5 nmol	L-017427-01-0005
si*DGAT1*	Dharmacon	SMARTpool	5 nmol	L-003922-00-0005
si*DGAT2*	Dharmacon	SMARTpool	5 nmol	L-009333-00-0005
si*MFSD2A*	Dharmacon	SMARTpool	20nmol	L-014999-02-0020

qPCR primer list

**Gene name**	**For-Primer**	**Rev-Primer**
SCREB1c	TGGACGAGCCACCCTTCAG	AGGGAAGTCACTGTCTTGGTTG
ACACA	TTGTTTGGTCGTGATTGCTCTG	ACAGGTATTCCACAGTCCCAG
FASN	CACTGCCGTGGAGAACATGC	GGATGCGGGAATACAGGTGAC
SCD	GCTGAGAAACTGGTGATGTTCC	GATATCCGAAGAGGTGGGCAG
PPARG1C	ACAGCTTTCTGGGTGGACTC	AGGGCAATCCGTCTTCATCC
PPARA	AAGCTGTCCTGGCTCAGATG	GATAGCCTGAGGCCTTGTCC
APOA1	GTGTCCCAGTTTGAAGGCTC	CTCCAGATCCTTGCTCATCTC
HMGCR	GCGTGGTGTATCTATTCGCCG	TGCCACTCCAACAGGGATGG
LDLR	CAAGTGGACTGCGACAACGG	CGAACTGCCGAGAGATGCAC
DGAT1	AGTGGCTTCAGCAACTACCG	GATGAGGTTCTCCAGAAATAACC
DGAT2	ATATTCTGCACTGATTGCTGGC	AGTTTCGGACCCACTGTGAC
NR1H4	TTCCCATTTACCTACCACAGATG	AGAGGACCTGCCACTTGTTC
FABP1	GCAAGTACCAACTGCAGAGC	CCCCCACCGTGAATTCGTTT
Mfsd2a	ACAAGCTTTGCTATGCAGTTGG	CAGAGGGTCAGTGAAGGCATC

Taqman assays

**Gene**	**Assay ID**
Chrebpbeta	AIVI4CH
Fasn	Mm00662319_m1
Scd1	Mm00772290_m1
Acly	Mm00652520_m1
Acaca	Mm01304285_m1
Srebp1c	Mm00550338_m1
Ppargc1a	Mm00447183_m1
Pppara	Mm00440939_m1
Apoa1	Mm00437568_g1
Apoa5	Mm04209850_g1
Apob	Mm01545159_m1
Apoc3	Mm00445670_m1
Hmgcr	Mm01282499_m1
Ldlr	Mm00440169_m1
Nr1h4	Mm00436419_m1
Dgat1	Mm00515643_m1
Dgat2	Mm00499530_m1
Angptl3	Mm00803820_m1
Angptl4	Mm00480431_m1
Cd36	Mm00432403_m1
Fabp1	Mm00444340_m1
Fabp4	Mm00445880_m1
Slc27a5	Mm00846873_g1
Gpam	Mm00833328_m1
Pnpla2	Mm00503040_m1
Ddit3	Mm00492097_m1
Tnf	Mm00443258_m1
Ccl2	Mm00446170_m1
Cxcl10	Mm00445235_m1
Slc2a2	Mm00446224_m1
Gapdh	Mm99999915_g1
Gck	Mm00439129_m1
G6pc	Mm00839363_m1
Col1a1	Mm00801666_g1

Supplemental Tables

Chr.	Genomic position	Gene	mRNA reference number	Nucleotide change	Amino acid alteration	gnomAD browser: MAF [%]	CADD (>20)	M-CAP (>0.025)	ClinPred (>0.5)	OMIM phenotype and MIM number
**17**	80039106	*FASN*	NM_004104.4	c.6529C>T	p.(Arg2177Cys)	0	24.7	0.041	0.993	–
**1**	22157724, mat.	*HSPG2*	NM_005529.5	c.11546G>C	p.(Arg3849Pro)	0.004604	24.0	0.117	0.064	DDSH, #224410; SJS1, #255800
**1**	22192236, pat.	*HSPG2*	NM_005529.5	c.4288C>T	p.(Leu1430Phe)	0.003186	23.5	0.030	0.845	DDSH, #224410; SJS1, #255800
**1**	86818601, pat.	*ODF2L*	NM_001007022.2	c.1834G>C	p.(Glu612Gln)	0.004730	27.5	0.014	0.508	–
**1**	86838152, mat.	*ODF2L*	NM_001007022.2	c.882A>T	p.(Glu294Asp)	0.01651	24.6	0.024	0.256	–

Table S1. *In silico* pathogenicity prediction, minor allele frequency, and associated OMIM phenotypes of *de novo* and biallelic variants in the patient

Trio-exome data were filtered for potentially pathogenic *de novo* variants absent in the general population (dbSNP138, 100 Genomes Project, Exome Variant Server, ExAC Browser, and gnomAD Browser) and rare biallelic and hemizygous variants with minor allele frequency (MAF) <0.1% and no homozygous or hemizygous carriers were found in the aforementioned databases. The functional impact of the identified variants from the trio exome was predicted by the Combined Annotation Dependent Depletion (CADD) tool, the Rare Exome Variant Ensemble Learner (REVEL) scoring system, the Mendelian Clinically Applicable Pathogenicity (M-CAP) Score, and the ClinPred tool. CADD is a

framework that integrates multiple annotations in one metric by contrasting variants that survived natural selection with simulated mutations. Reported CADD scores are phred-like rank scores based on the rank of that variant's score among all possible single nucleotide variants of hg19, with 10 corresponding to the top 10%, 20 at the top 1%, and 30 at the top 0.1%. The larger the score the more likely the variant has deleterious effects; the score range observed here is strongly supportive of pathogenicity, with all observed variants ranking above ~99% of all variants in a typical genome and scoring similarly to variants reported in ClinVar as pathogenic (~85% of which score >15).[1] M-CAP is a classifier for rare missense variants in the human genome, which combines previous pathogenicity scores (including SIFT, Polyphen-2, and CADD), amino acid conservation features and computed scores trained on mutations linked to Mendelian diseases. The recommended pathogenicity threshold is >0.025. ClinPred is an ensemble classifier for predicting disease relevance of missense variants, combining random forest and gradient boosting models and employing allele frequencies of the variants in different populations from the gnomAD database, with a score ranging from 0-1. The higher the score the more likely the variant is pathogenic. The recommended pathogenicity threshold is ≥ 0.5 The *de novo* FASN variant was absent in gnomAd and showed predictions higher than the indicated thresholds. Some nearby variants are also found in the general population, such as p.Arg2180Gln (gnomAd frequency of 0.00003). Loss-of-function FASN variants

are available in open databases such as gnomAd and the UK Biobank suggesting probably an incomplete penetrance, a variable expressivity with e.g. some milder or more late-onset phenotypes and/or the specific dominant negative variant effect of this variant (Fig.1). Whether loss of function variants in these databases or other variants listed in ClinVar might be possibly linked to a FASN-associated disease, remains to be shown in future studies investigating more patients with variants in FASN. DDSH = Dyssegmental dysplasia, Silverman-Handmaker type; SJS1 = Schwartz-Jampel syndrome type 1; Chr. = chromosome; MAF = minor allele frequency; mat. = maternal; pat. = paternal; – = not available.

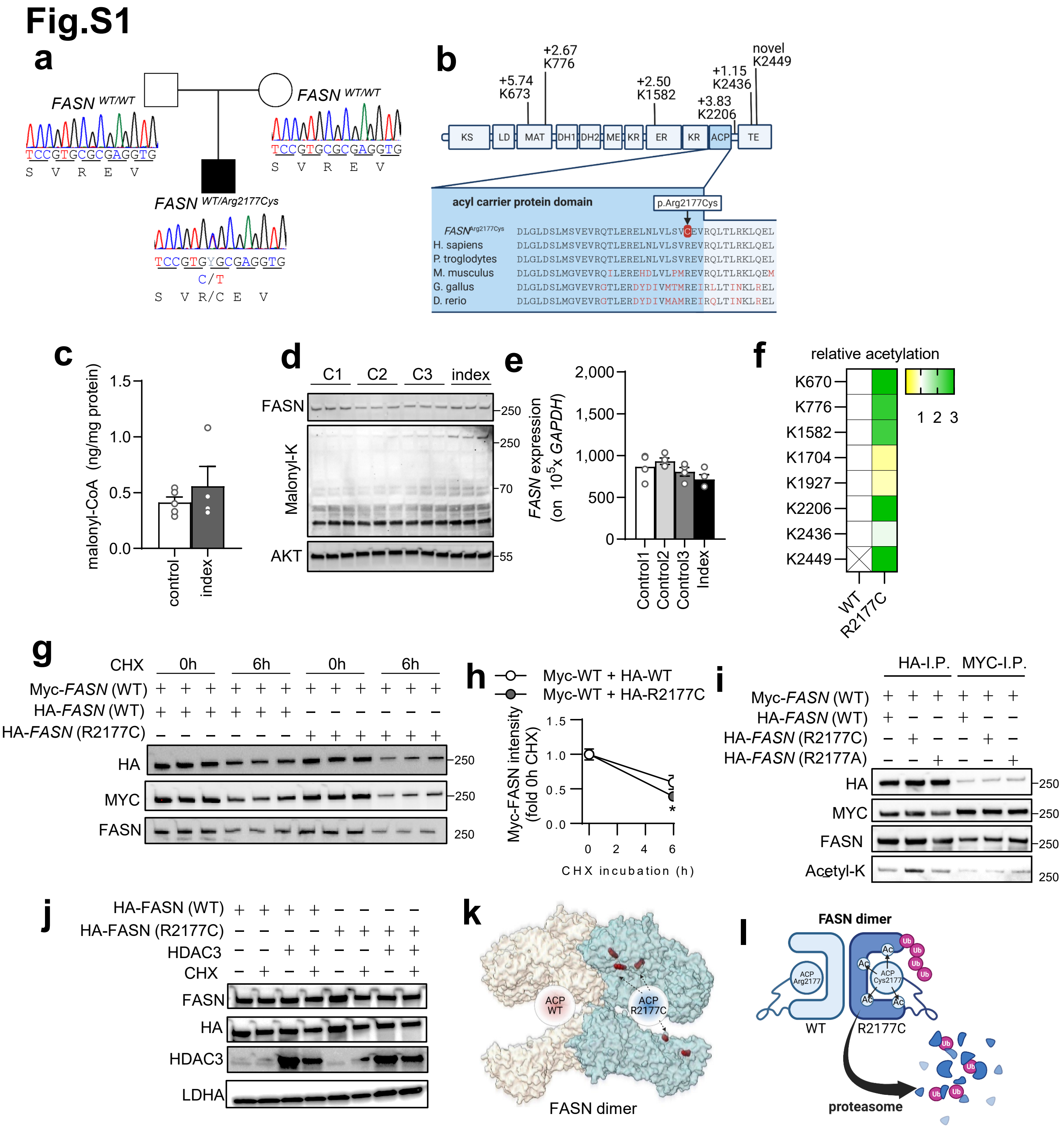

Fig.S1: Functional analysis of a FASN^{Arg2177Cys} variant. (a) Pedigree tree of the affected family indicating a heterozygous de novo variant in *FASN* [c.6529C>T (p.Arg2177Cys)] and wild type (WT) *FASN* loci of the parents found by trio-whole exome sequencing with sequencing traces of confirmative sanger sequencing of parents and index patient indicating a de novo missense mutation in *FASN*. (b) Localization of missense variant in the ACP domain and conservation of the amino acid. K indicates mass spec-defined acetyl-lysine sites and fold induction of the positively altered acetylation sites is indicated. (c) Malonyl-CoA levels and (d) western blot of protein malonylation of index fibroblasts. (e) *FASN* gene expression in index fibroblasts and controls. (f) relative acetylation levels of WT and R2177C FASN variant overexpressed and immunoprecipitated from HEK293T cells and analyzed via mass spectrometry. (g) Western blot and (h) quantification of HEK cells, which were contransfected with the indicated constructs to delineate a dominant negative effect of R2177C-FASN on protein stability in a CHX assay. (i) Western blot against MYC, HA, FASN, and Acetely-K of HEK cell cotransfected with indicated constructs (WT/R2177C/R2177A) to assess potential transacetylation of the WT variant. (j) WT and R2177CFASN overexpression with coexpression of HDAC3 or empty vector with or without 6h cycloheximid (CHX) incubation. (k)Computational structural model of a putative WT and FASN^{Arg2177Cys} dimer. Hyperacetylated lysines are marked in red. (l) Proposed model of the protein instability. FASN^{Arg2177Cys} is non-enzymatically acetylated by the missense cysteine, which is recognized and ubiquitinylated by E3-ligases and further degraded by the proteasome. Error bars are indicating standard error of the mean (SEM).

Fig.S2

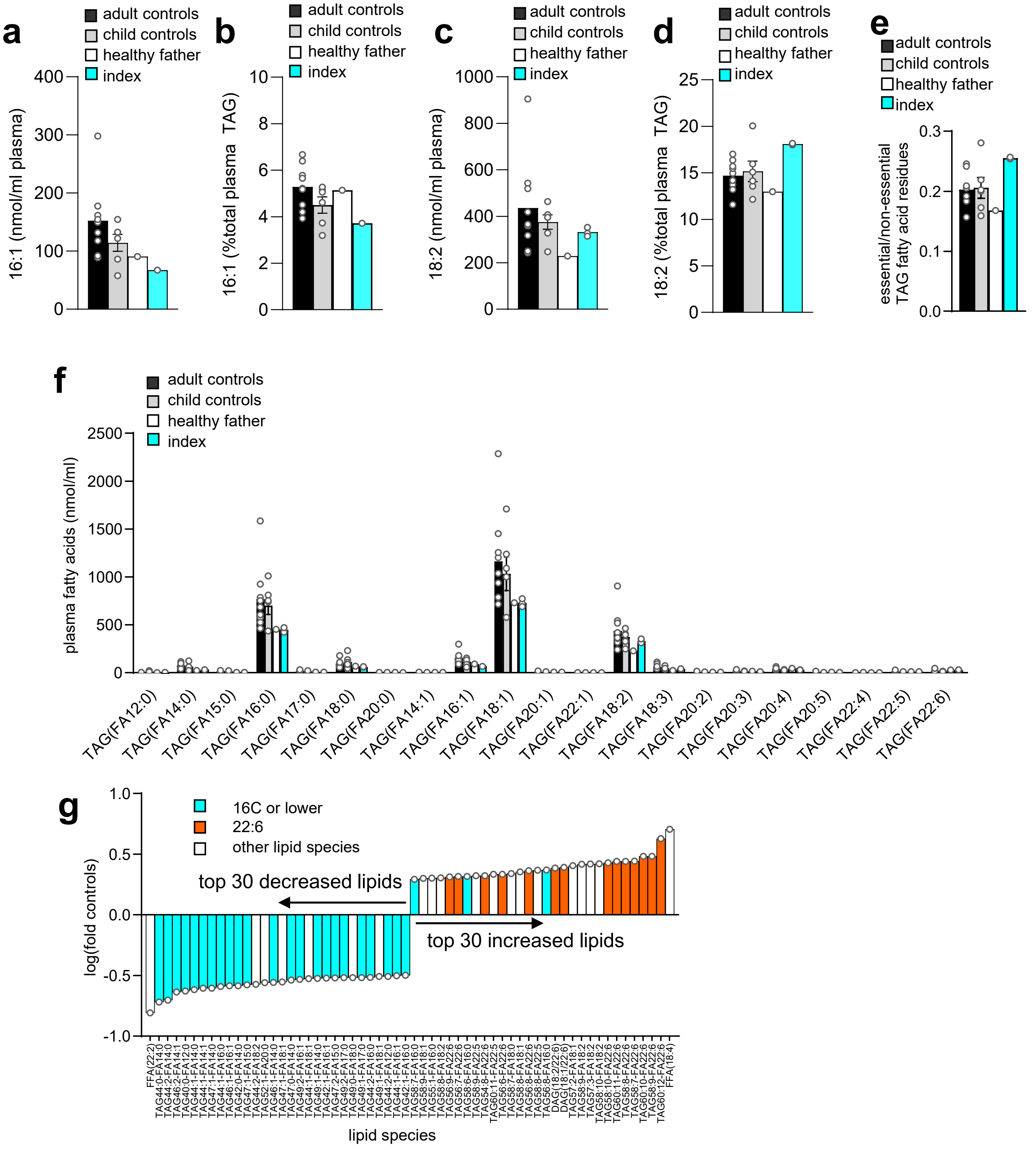

Fig.S2: Plasma Lipidomics of genetic FASN modulation. Panel (a-d) show the absolute and relative values, which have been used to calculate the lipogenic index in Fig.2a (adult controls *n*=9, child controls *n*=6, father, index *n*=1). (e) Plot of essential vs. non-essential TAG fatty acid residues. (f) Concentration of plasma TAG species measured with LC/MS. Data are shown as mean (bars) and error bars indicate standard error of the mean (SEM). (g) Plot of top 30 increased/decreased lipids in the index patients' plasma (log-transformed fold induction of all investigated lipid species compared to the mean of healthy control patients). "16C or lower" indicates all lipid species with fatty acid residues of 16 carbon atoms or lower (independent of desaturation) and 22:6 indicates existing docosahexaenoic acid residues within the investigated lipid (*n*=1).

Fig.S3

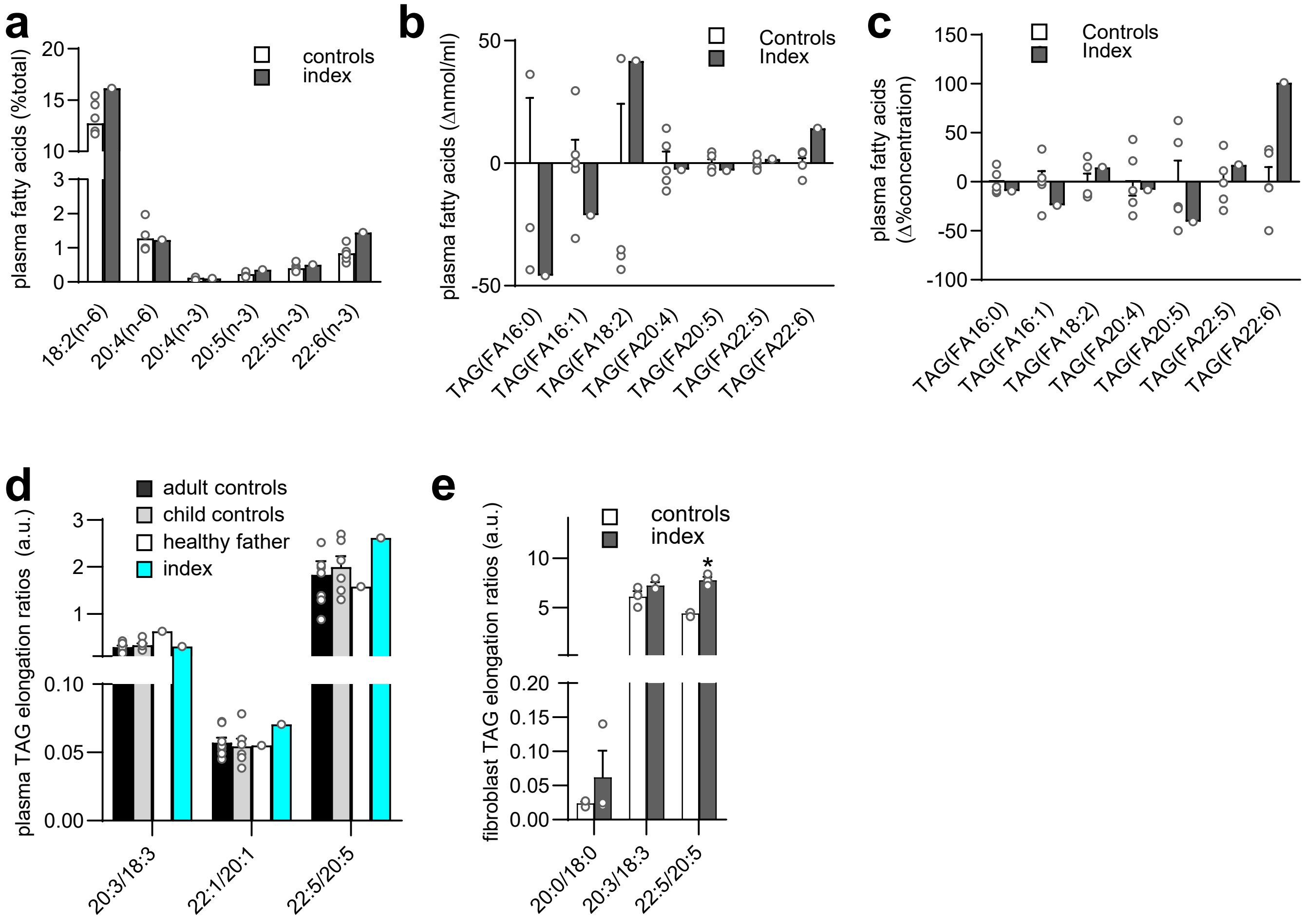

Fig.S3: Lipidomics of a patient carrying a de novo FASN variant. (a) GC/MS fatty acid profile measurements of index (*n*=1) and control plasma (*n*=9) to quantify omega-3 and omega-6 fatty acids. (b) Absolute and (c) relative delta of indicated fatty acid species between index (*n*=1) and control (*n*=5) plasma in LC/MS measurements. (d) LC/MS-based ratios in TAG species of plasma from adult controls (*n*=9), child controls (*n*=6), the healthy father (*n*=1) and the index patient (*n*=1) and (e) LC/MS-based ratios in TAG species of fibroblasts from controls (n=3) and the index patient (*n*=3) indicating the elongation of several fatty acid species found in the samples. Data are shown as mean (bar) and error bars indicate standard error of the mean (SEM).

Fig.S4

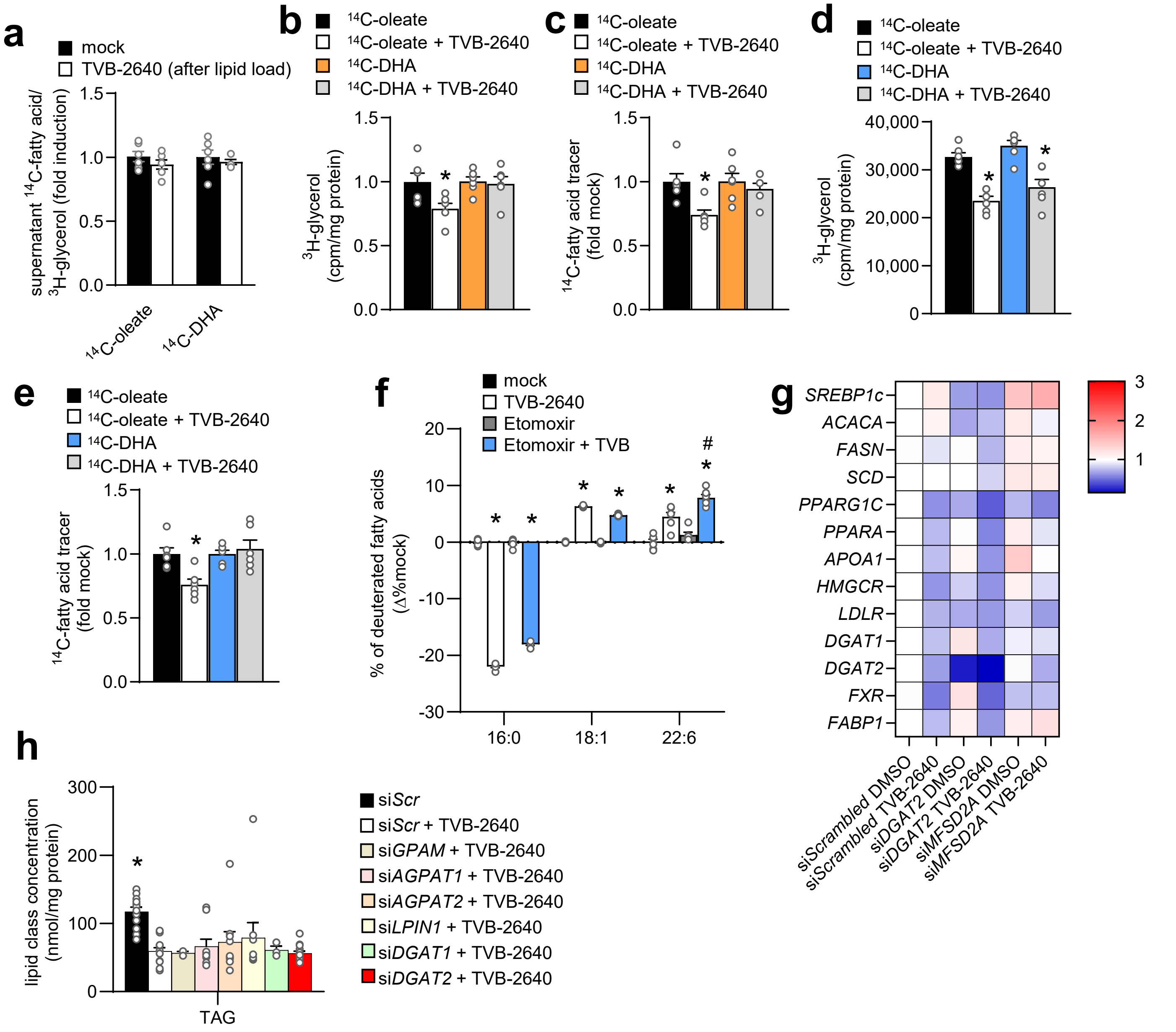

Fig.S4: Pharmacologic inhibition of FASN in HuH7 cells. (a-c) To stimulate lipoprotein secretion, HuH7 cells were loaded with oleate, and DHA mixed with radioactively labeled tracers overnight. After lipid loading, cells were extensively washed and incubated with TVB-2640 or control. Supernatant was collected 5h after inhibition and lipid extract was counted via scintillation counting. (a) supernatant ^{14}C-fatty acid counts per ^{3}H-glycerol counts (*n*=6) (b) ^{3}H-glycerol or (c) ^{14}C-fatty acid tracer counts in supernatant of ^{14}C-oleate/^{3}H Glycerol respectively ^{14}C-DHA/^{3}H-glycerol treated cells with or without FASN inhibitor TVB-2640 after or (d+e) during radioactive lipid load. (*n*=6) (f) Incubation of HuH7 cells with equimolar doses of deuterated palmitate, oleate and DHA. (*n*=6) (g) Gene expression (*n*=6) and (h) TAG concentration of cells treated with indicated siRNA and/or TVB-2640 (*n*=12 for siScr, *n*=12 for siScr + TVB-2640, *n*=3 for si*GPAM* + TVB-2640, *n*=9 for si*GPAT1* + TVB-2640, *n*=9 for si*GPAT2* + TVB-2640, *n*=9 for si*LPIN1* + TVB-2640, *n*=3 for si*DGAT1* + TVB-2640, *n*=15 for si*DGAT2* + TVB-2640). (*) indicates *P*-value of <0.05 in comparison to the respective control group (respectively vs. SiScr + TVB-2640 in panel h, white bar) or (#) indicates *P*-value of <0.05 in 22:6 vs respective group in 16:0 and 18:1 (panel f) calculated via two-way ANOVA and Fisher's LSD test. Error bars are indicating standard error of the mean (SEM).

Fig.S5

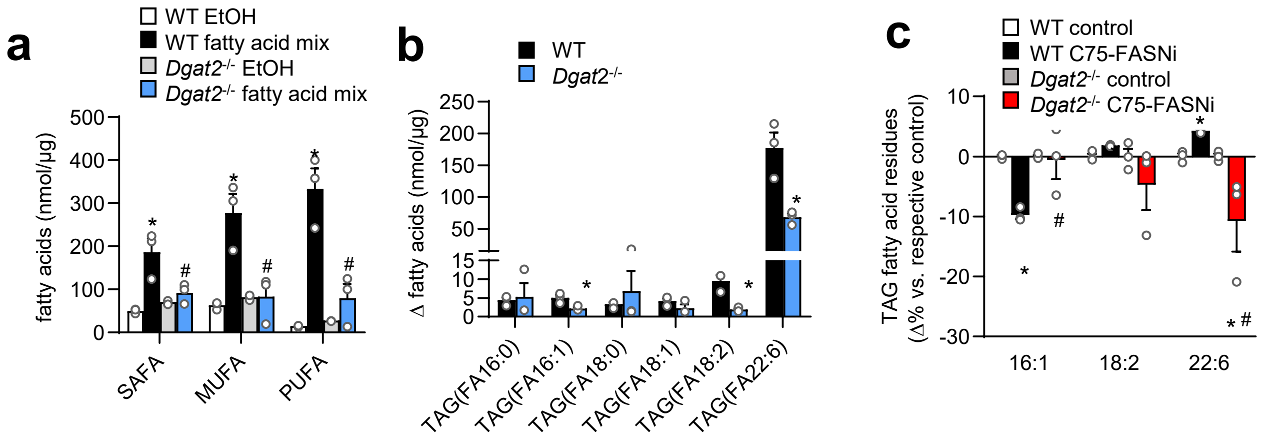

Fig.S5: Selective PUFA incorporation is regulated by FASN and DGAT2. (a) Absolute concentration of fatty acid residues in TAG of wild type and *Dgat2*$^{-/-}$ mouse embryonic fibroblasts (MEFs) incubated with either control (EtOH) (WT EtOH: *n*=3; *Dgat2*$^{-/-}$ EtOH: *n*=3) or an equimolar fatty acid mix of 16:0/16:1/18:0/18:1/18:2/22:6 (WT fatty acid mix: *n*=3; *Dgat2*$^{-/-}$ fatty acid mix EtOH *n*=3) (b) Absolute concentrations of selected fatty acids in WT and *Dgat2*$^{-/-}$ MEFs incubated with the equimolar fatty acid mix as indicated in (a) (*n*=3). (c) Wild type and *Dgat2*$^{-/-}$ MEFs incubated with the equimolar fatty acid mix indicated in panel (a) with or without the fatty acid inhibitor C75 (C75-FASN-i) or saline (control) (WT control: *n*=3; WT C75-FASN-i: *n*=3; *Dgat2*$^{-/-}$ control: *n*=3; *Dgat2*$^{-/-}$ C75-FASN-i: *n*=3). Data are shown as mean (bars) and error bars show standard error of the mean (SEM). (*) indicates statistical significance of p<0.05 versus the respective control treated and (#) indicates statistical significance of p<0.05 versus the repective WT group and was calculated by using two-way ANOVA uncorrected Fisher's LSD Test (a, c) or Students T-Test, adjusted with Holm-Sidak method for multiple testing (b).

Fig.S6

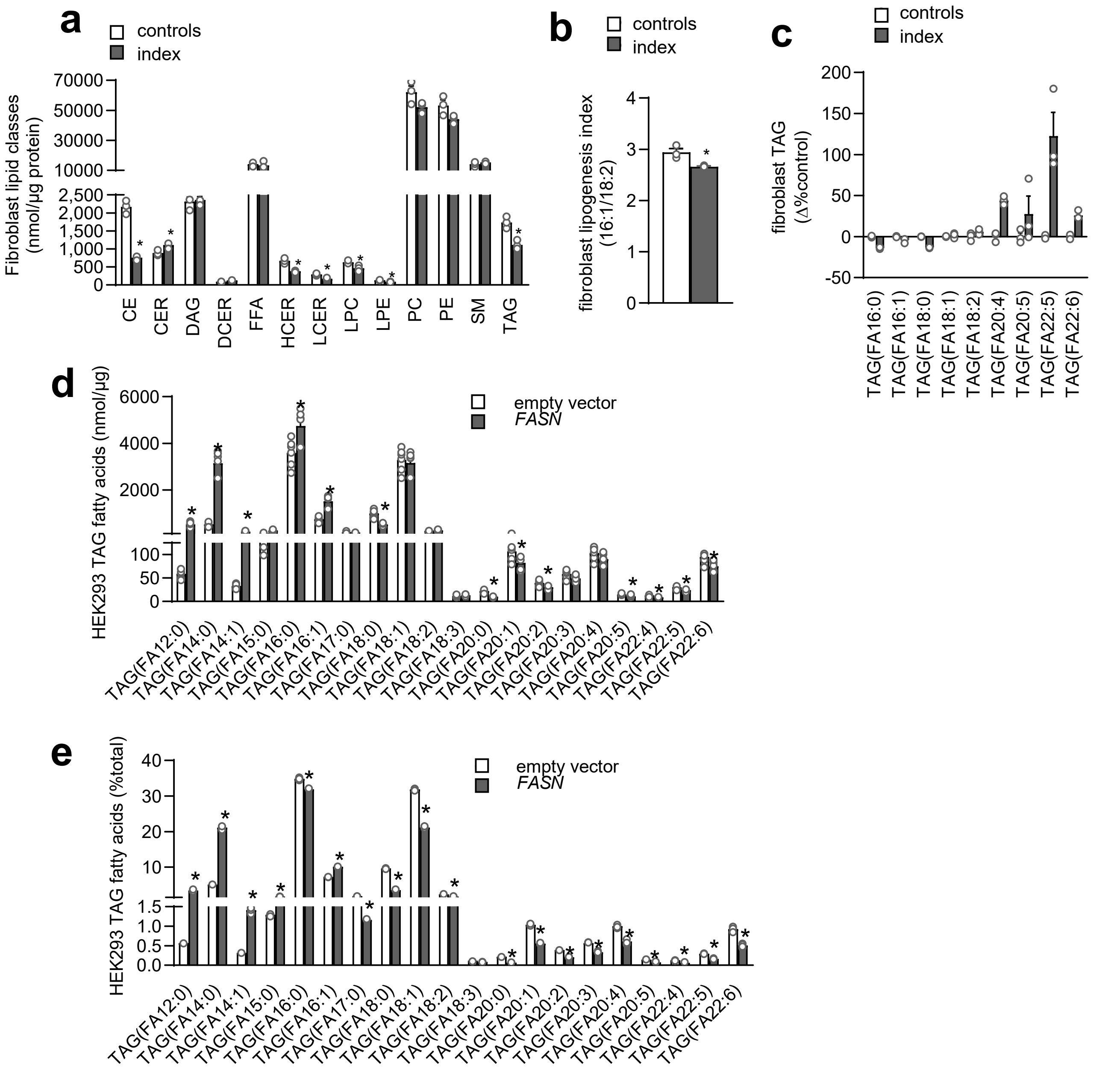

Fig.S6: Lipidomics of index fibroblasts or HEK cells overexpressing FASN. (a) Lipidomic analysis of control (control: *n*=3) or index fibro- blasts (index: n=3) of lipid classes choelsterylester (CE), ceramides (CER), diacylglycerols (DAG), dihydroceramides (DCER), free fatty acids (FFA), hexosylceramides (HCER), lactosylceramides (LCER), lysophosphatidylcholines (LPC), lysophosphatidylethanolamines (LPE), phosphatidylcholines (PC), phosphatidylethanolamines (PE), sphingomyelins (SM), triacylglycerols (TAG). (b) Lipogenesis index in index (index: n=3) or control fibroblasts (control: *n*=3) . (c) Delta of TAG PUFA species composition of index (index: n=3) or control fibroblasts (control: *n*=3) . (d) Concentration or (e) composition of TAG fatty acid species in HEK cells overexpressing control (empty vector *n*=6) or FASN *(n*=6). Data are shown as mean (bar) and error bars indicate standard error of the mean (SEM). (*) indicates statistical significance of p<0.05 versus the respective controls and was calculated by using Students T-Test.

Fig.S7

a

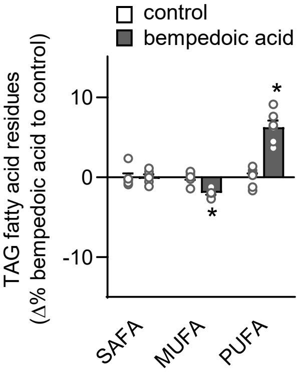

Fig.S7: Alternative pharmacologic approach targeting *de novo* lipogenesis. SAFA, MUFA and PUFA of TAG in HuH7 cells treated with control (control *n*=6) or bempedoic acid *(n*=6). Data are shown as mean (bar) and error bars indicate standard error of the mean (SEM).(*) indicates statistical significance of $p<0.05$ versus the respective controls and was calculated by using Students T-Test.

Fig.S8

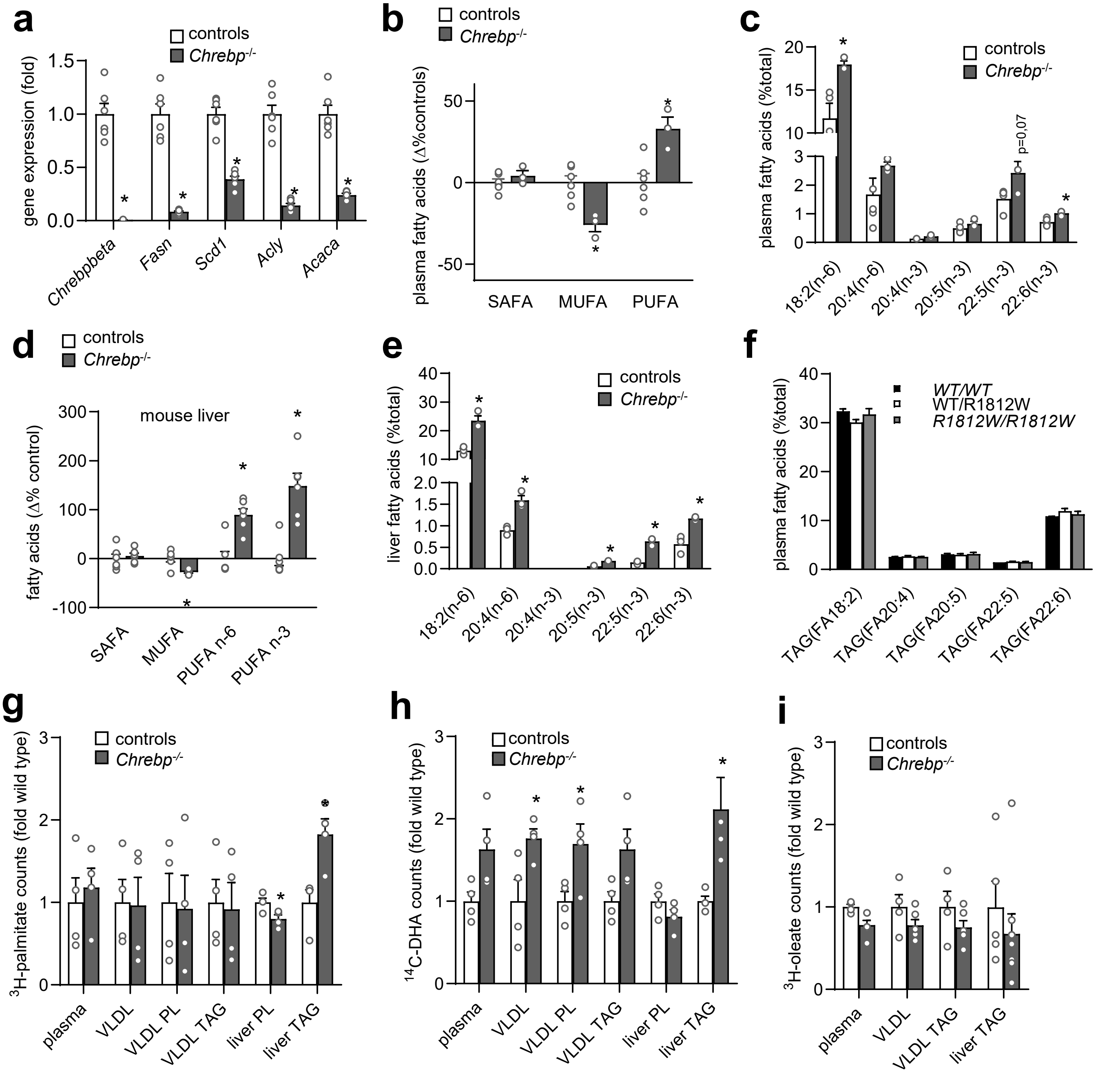

Fig.S8: *$Chrebp^{-/-}$* mice, a mouse model of low lipogenesis, phenocopy the higher PUFA abundance. (a) Liver gene expression of male wild type (controls *n*=6) or male *$Chrebp^{-/-}$* mice (*$Chrebp^{-/-}$* *n*=6). (b) Plasma fatty acid composition assessed by LC-MS of female wild type (controls *n*=6) and female *$Chrebp^{-/-}$* mice (*$Chrebp^{-/-}$* *n*=3). (c) GC-FiD analysis of different n-6 and n-3 PUFA species in TAG fraction isolated via thin layer chromatography of female wild type (control *n*=5) and female *$Chrebp^{-/-}$* mice (*$Chrebp^{-/-}$* *n*=3). (d-e) Liver fatty acid composition of different n-6 and n-3 PUFA species in TAG fraction isolated via thin layer chromatography of male wild type (controls *n*=6) and male *$Chrebp^{-/-}$* mice (*$Chrebp^{-/-}$* *n*=6). (f) LC-MS-based plasma fatty acid composition analysis of TAG of male and female wild type *(WT/WT n*=4), male and female heterozygous (*WT/R1812W n*=6) or male and female homozygous FASN-R1812W (*R1812W/R1812W n*=7) transgenic mice. (g-i) Tracer counts of male WT (WT) or male *$Chrebp^{-/-}$* (*$Chrebp^{-/-}$*) mice injected with radioactively labelled palmitate (g: WT *n*=4; *$Chrebp^{-/-}$* *n*=4) , DHA (h: WT *n*=4; *$Chrebp^{-/-}$* *n*=4) or oleate (i: WT *n*=4-5; *$Chrebp^{-/-}$* *n*=5-8) in the respective liver or plasma fractions. Data are shown as mean (bar) and error bars indicate standard error of the mean (SEM). * indicates $p<0.05$ by calculation of Student's *t*-test .

Fig.S9

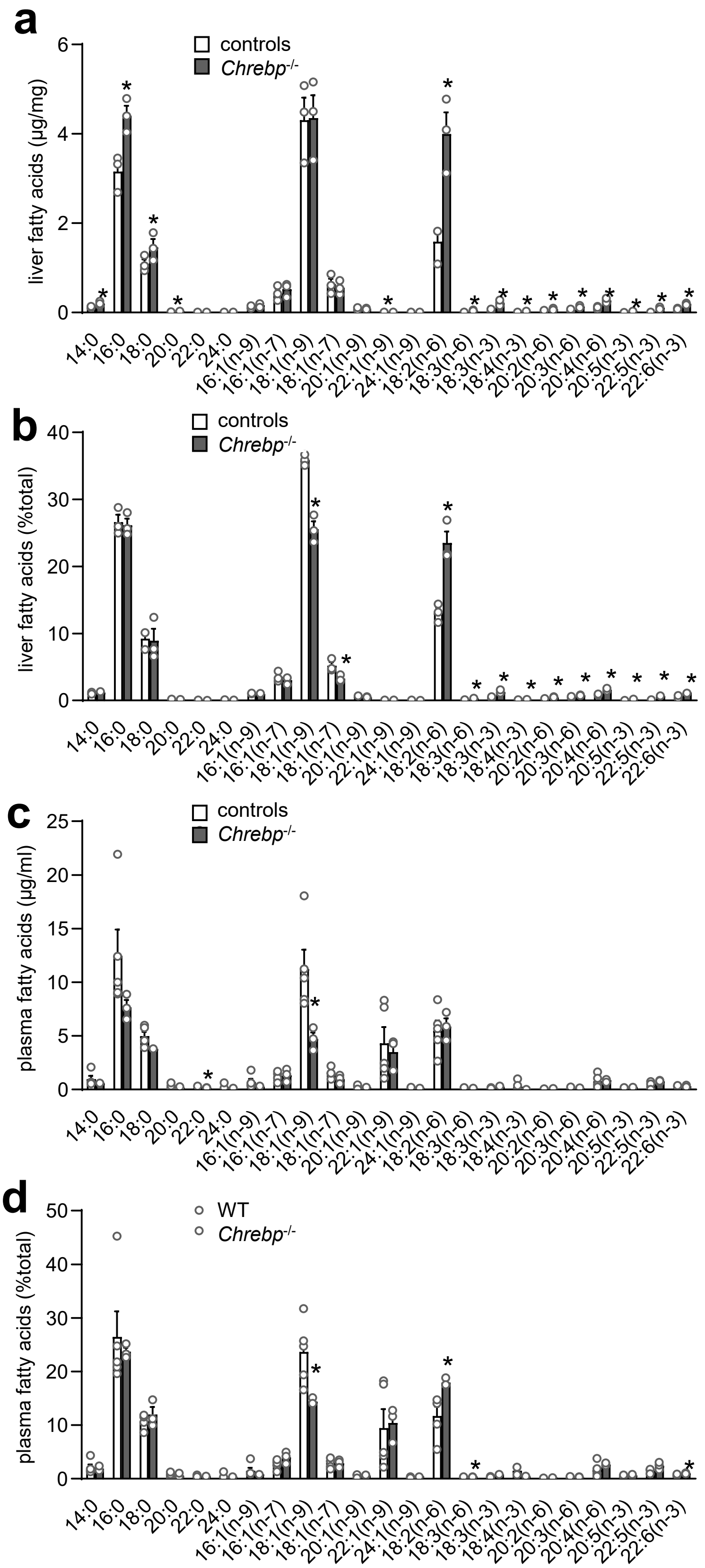

Fig.S9: Fatty acid profiles from *Chrebp$^{-/-}$* mice. (a) Absolute and (b) relative fatty acid profiles from livers of male *Chrebp$^{-/-}$* mice (*Chrebp$^{-/-}$* mice *n=3)* or male wild type littermates (WT *n*=3) and (c+d) plasma from female *Chrebp$^{-/-}$* mice (*Chrebp$^{-/-}$ n*=3) or female wild type littermates (WT *n*=5). Data are shown as mean (bar) and error bars indicate standard error of the mean (SEM). (*) indicates p<0.05 by calculation of Students *t*-Test.

Fig.S10

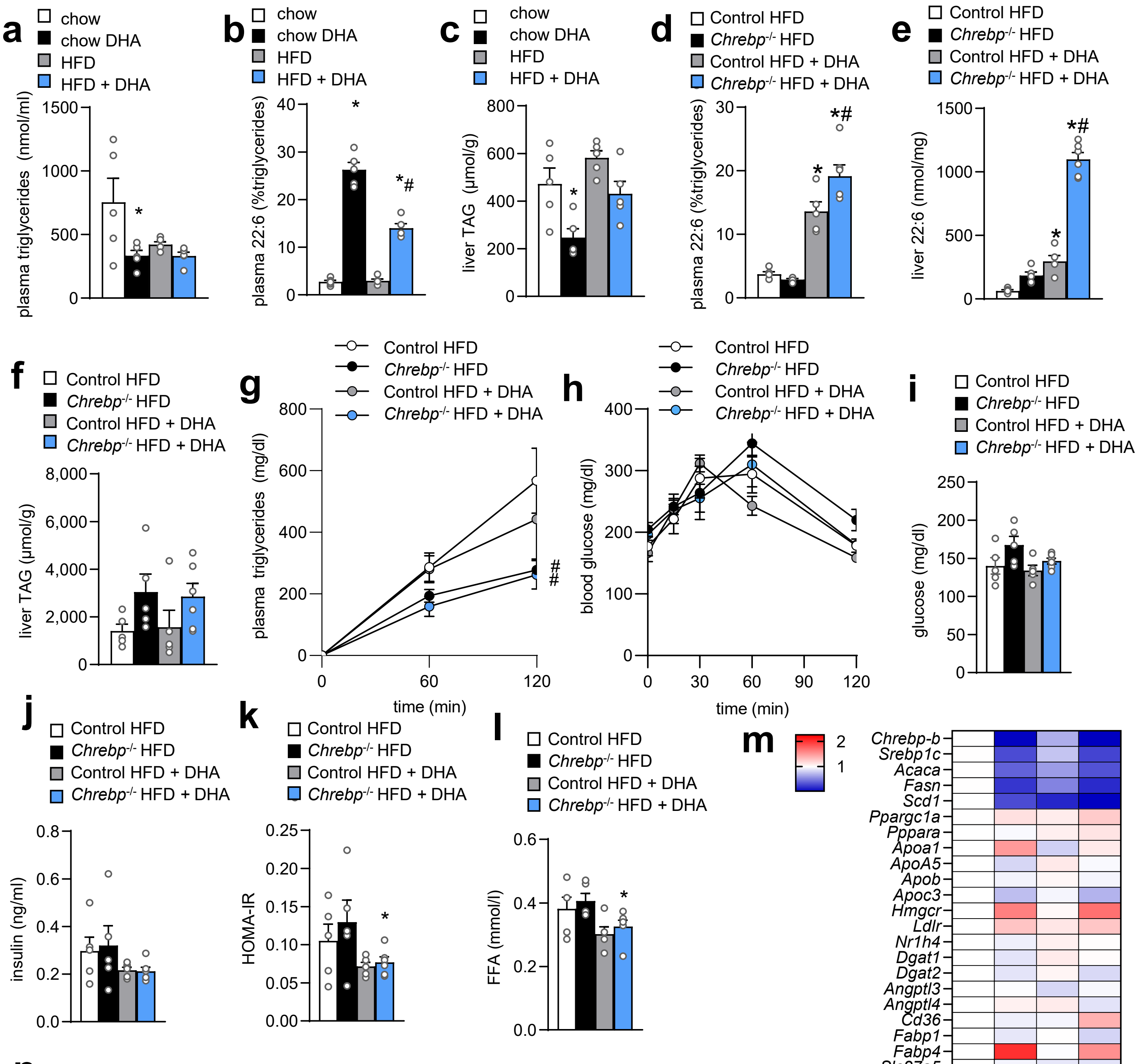

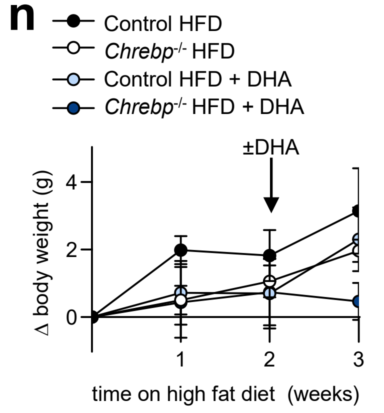

Fig.S10: DHA supplementation in *Chrebp*$^{-/-}$ mice. (a) Plasma triglycerides, (b) plasma DHA and (c) liver triglycerides in male wild type mice supplemented with DHA on chow and HFD (chow *n*=5, chow DHA *n*=5, HFD *n*=5, HFD DHA *n*=5). (d) plasma DHA, (e) liver DHA, (f) liver TAG in HFD-fed female WT and *Chrebp*$^{-/-}$ mice (control HFD *n*=5; *Chrebp*$^{-/-}$ HFD *n*=5; Control HFD + DHA *n*=5; *Chrebp*$^{-/-}$ HFD + DHA , *n*=6) (g) vldl secretion in male HFD-fed WT and *Chrebp*$^{-/-}$ mice (control HFD *n*=5; *Chrebp*$^{-/-}$ HFD *n*=5; Control HFD + DHA *n*=5; *Chrebp*$^{-/-}$ HFD + DHA , *n*=5) (h) oral glucose tolerance test in male HFD-fed WT and *Chrebp*$^{-/-}$ mice (control HFD *n*=5; *Chrebp*$^{-/-}$ HFD *n*=5; Control HFD + DHA *n*=6; *Chrebp*$^{-/-}$ HFD + DHA , *n*=6) (i) basal glucose (j) insulin, (k) HOMA-IR; (l) plasma free fatty acids, (m) liver gene expression and (n) body weigth curve of female wild type or *Chrebp*$^{-/-}$ mice fed a HFD for two weeks with an additional week of DHA supplementation or not. (*n*=5 for control HFD, *Chrebp*$^{-/-}$ HFD, Control HFD + DHA, *n*=6 for *Chrebp*$^{-/-}$ HFD + DHA). Data are shown as mean (bars) and error bars indicate standard error of the mean (SEM), significant differences were assessed using two way ANOVA and Fisher's LSD test with (*) = p<0.05 showing DHA effects vs. respective controls and (#) p<0.05 indicating diet (a-c) or genotpye (d-l) effects vs. respective controls.

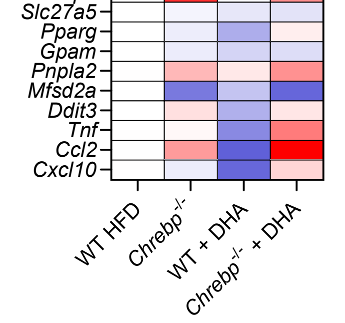

Fig.S11

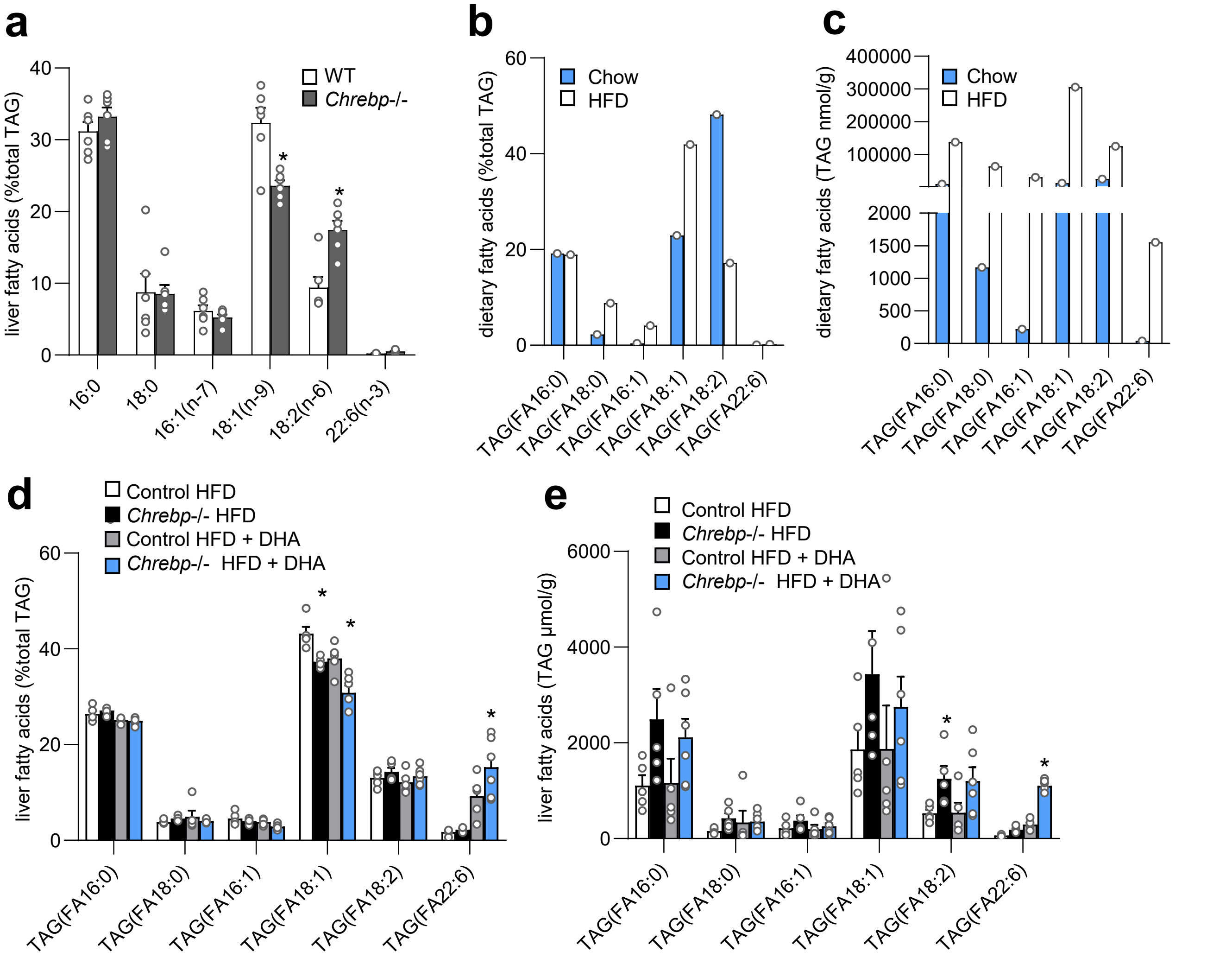

Fig.S11: Relation of fatty acid profiles of livers of *Chrebp*$^{-/-}$ mice to the respective diet. (a) Fatty acid profile of livers of male wild type (WT *n*=6) and *Chrebp*$^{-/-}$ mice (*Chrebp*$^{-/-}$ *n*=6) on chow diet, (b) Composition and (c) concentration of TAG fatty acids from chow diet (chow *n*=1) and HFD (HFD *n*=1), (d) Composition and (e) concentration of hepatic TAG fatty acids of HFD fed female WT control and *Chrebp*$^{-/-}$ mice supplemented or not with DHA (*n*=5 for control HFD, *Chrebp*$^{-/-}$ HFD, Control HFD + DHA, *n*=6 for *Chrebp*$^{-/-}$ HFD + DHA). Data are shown as mean (bars) and error bars indcate standard error of the mean (SEM). Significant differences were assessed using Student's *t*-Test (a) or two way ANOVA and Fisher's LSD test (d,e) with (*) = p<0.05 showing genotype effects vs. respective controls.

Fig.S12

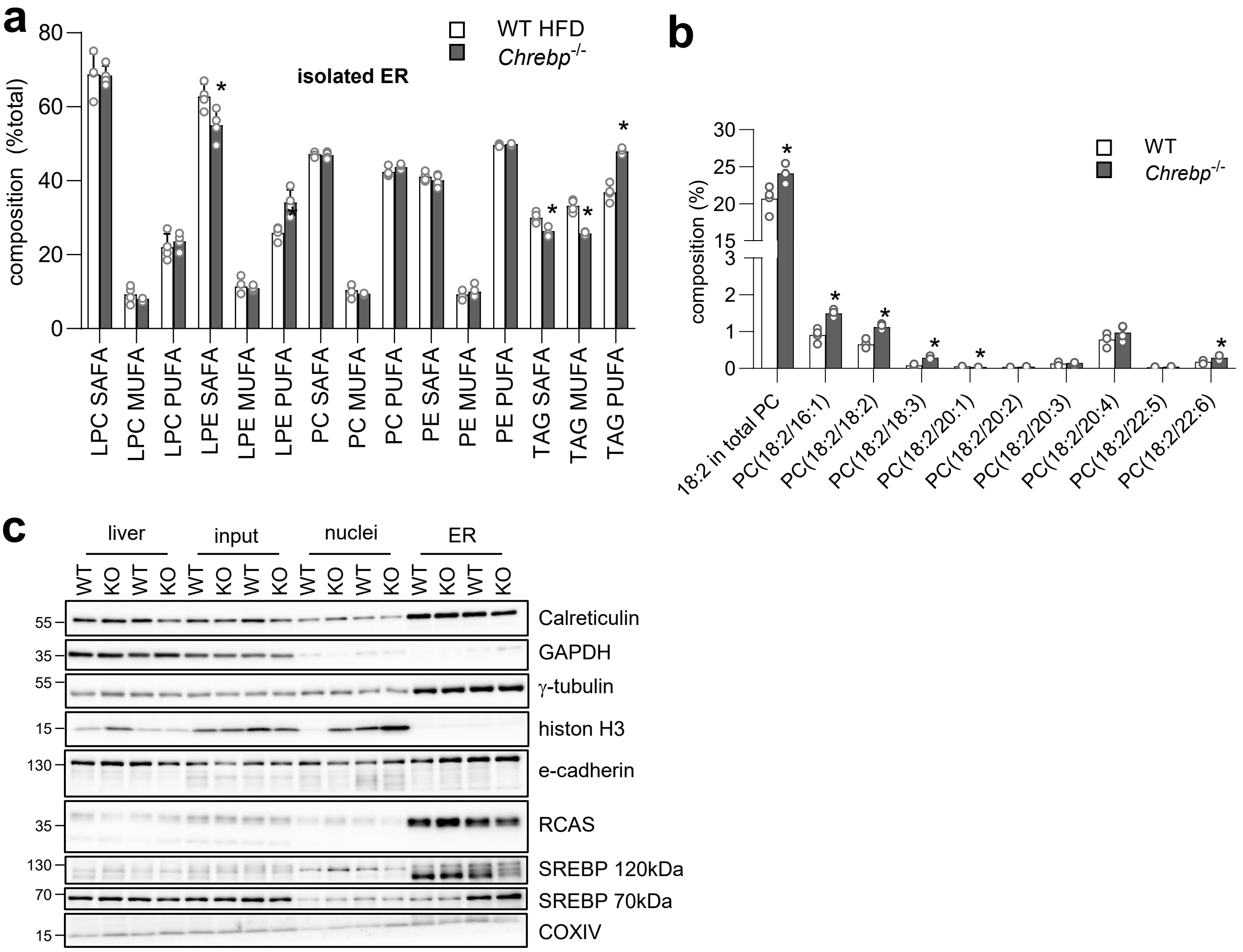

Fig.S12: Analysis of endoplasmic reticulum of wild type and *Chrebp*$^{-/-}$ mice. (a-b) Lipidomics of isolated endoplasmic reticulum of male wild type (WT *n=4)* and *Chrebp*$^{-/-}$ (*Chrebp*$^{-/-}$ *n*=4) mice and (c) western blot of isolated endoplasmic reticulum of male wild type (WT *n=2)* and *Chrebp*$^{-/-}$ (*Chrebp*$^{-/-}$ *n*=2) mice, indicated molecular weights in kDa.. Data are shown as mean (bars) and error bars indicate standard error of the mean (SEM) * indicates $p<0.05$ by calculation of Students *t-Test*.

Fig.S13

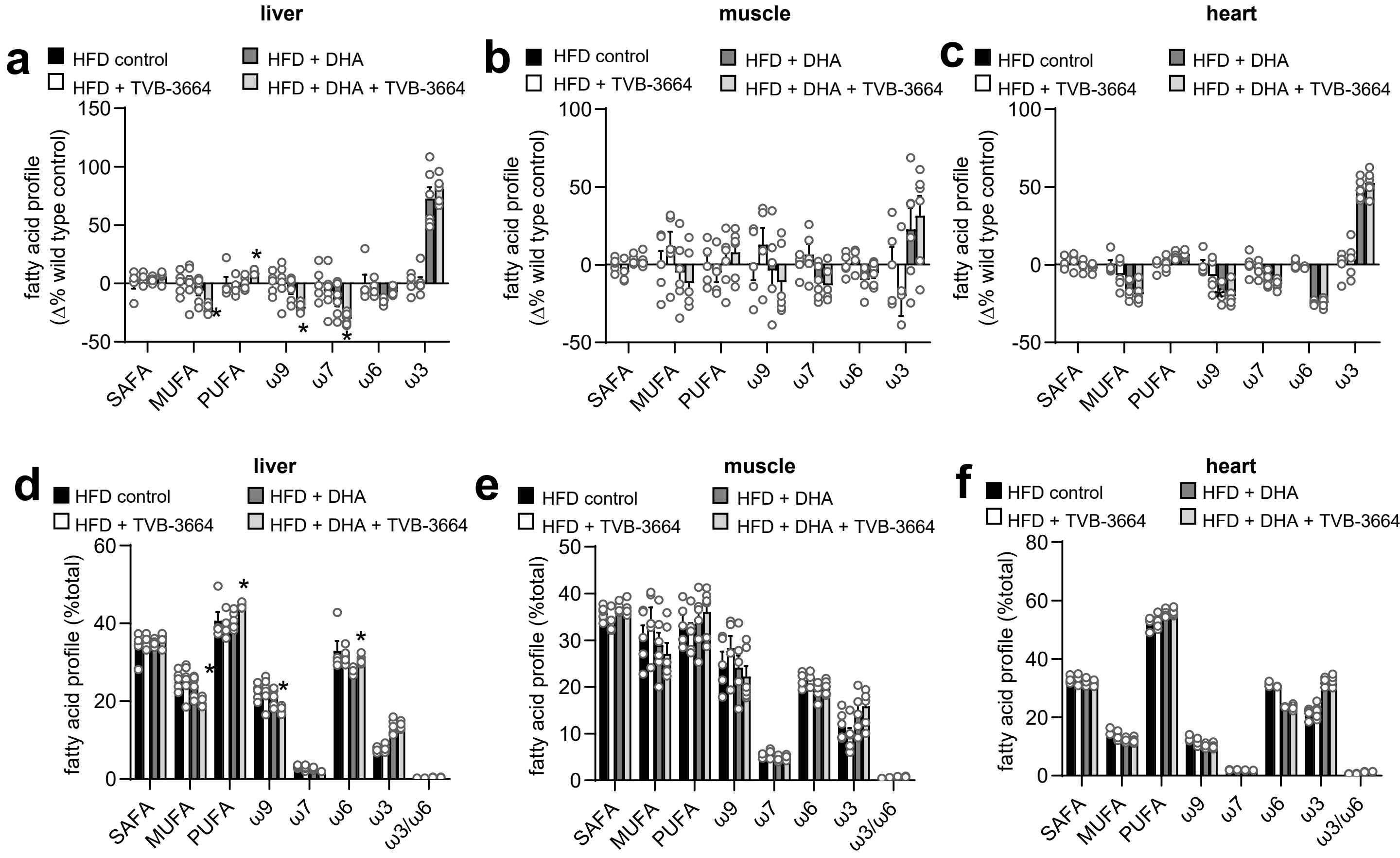

Fig.S13: Pharmacologic FASN inhibition *in vivo*. Differences in composition of fatty acids of (a) liver, (b) muscle, (c) heart and the relative free fatty acid profile (d,e,f) of male mice fed two weeks high fat diet (HFD) and an additional week with or without DHA supplementation and/or FASN inhibition with mouse FASN inhibitor TVB-3664 (HFD control *n*=5; HFD +TVB-3664 *n*=6 (a,d) *n*=5 (b,c,e,f); HFD + DHA *n*=6, HFD + DHA + TVB-3664 *n*=6). Data are shown as mean (bars) and error bars indicate standard error of the mean (SEM) * indicates p<0.05 of TVB-3664 group vs its respective non-TVB-3664 treated group calculated by two-way ANOVA and Fisher‘s LSD test.

Fig.S14

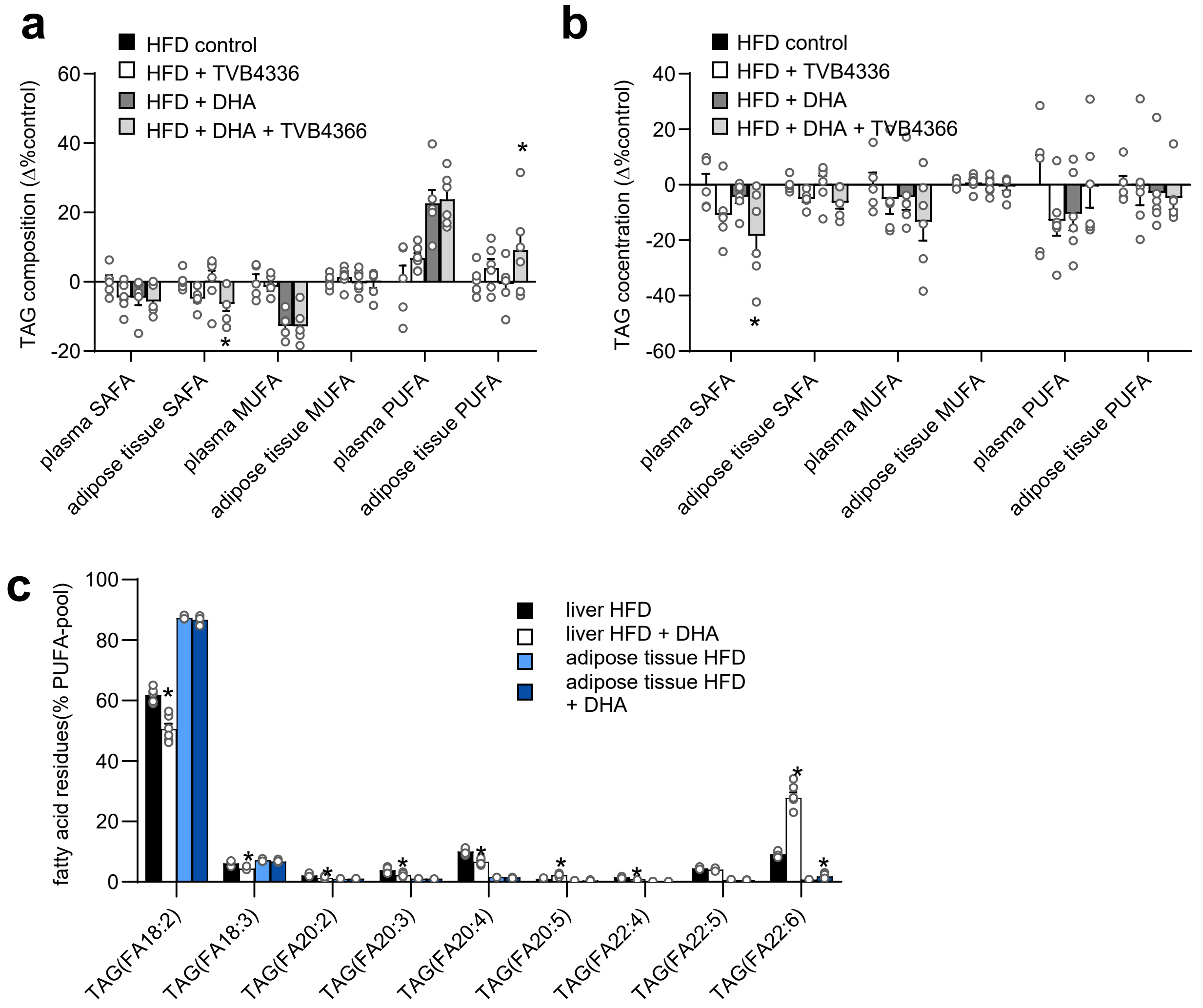

Fig.S14: Pharmacologic FASN inhibition *in vivo*. Differences in composition of fatty acids of (a) plasma and adipose tissue in TAG composition or (b) concentration of male mice fed two weeks high fat diet and an additional week with or without DHA supplementation and/or FASN inhibition with mouse FASN inhibitor TVB-3664 (HFD control *n*=5; HFD +TVB-3664 *n*=6; HFD + DHA *n*=6; HFD + DHA + TVB-3664 *n*=6) . (c) Comparison of liver and adipose tissue PUFA pool and effect of supplementation of DHA in vivo in male mice fed two weeks high fat diet and an additional week with or without DHA supplementation (HFD *n*=5; HFD + DHA *n*=6). Data are shown as mean (bars) and error bars indicate standard error of the mean * indicates p<0.05 of TVB-3664 group vs its respective non-TVB-3664 treated group (a-b) and DHA vs non DHA treated mice (c) calculated by two-way ANOVA and Fisher‘s LSD test.

Fig.S15

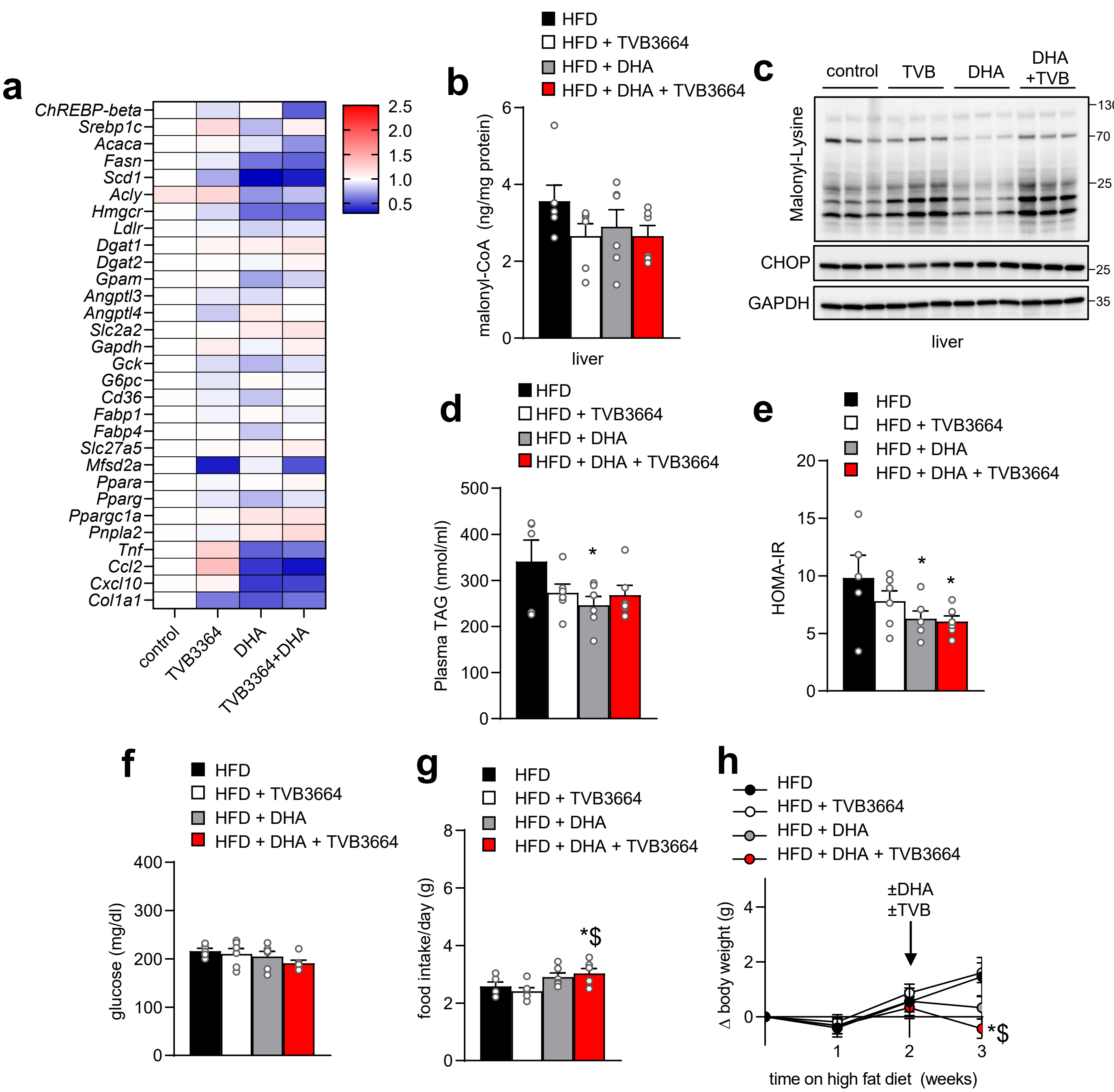

Fig.S15: Pharmacologic FASN inhibition *in vivo*. (a) gene expression, (b) malonyl-CoA, (c) western blot (HFD control *n*=3; HFD +TVB-3664 *n*=3; HFD + DHA *n*=3; HFD + DHA + TVB-3664 *n*=3), indicated molecular sizes in kDa (d) plasma TAG, (e) HOMA-IR, (f) basal glucose levels, (g) food intake and (h) body weight curve of male mice fed two weeks high fat diet and an additional week with or without DHA supplementation and/or FASN inhibition with mouse FASN inhibitor TVB-3664 (HFD control *n*=5; HFD +TVB-3664 *n*=6; HFD + DHA *n*=6; HFD + DHA + TVB-3664 *n*=6). Data are shown as mean (bars) and error bars indicate standard error of the mean, significant differences were assessed using two way ANOVA and Fisher's LSD test with (*) = p<0.05 showing significant differences vs. HFD and ($) showing significant differences vs. HFD+ TVB3664.

Fig.S16

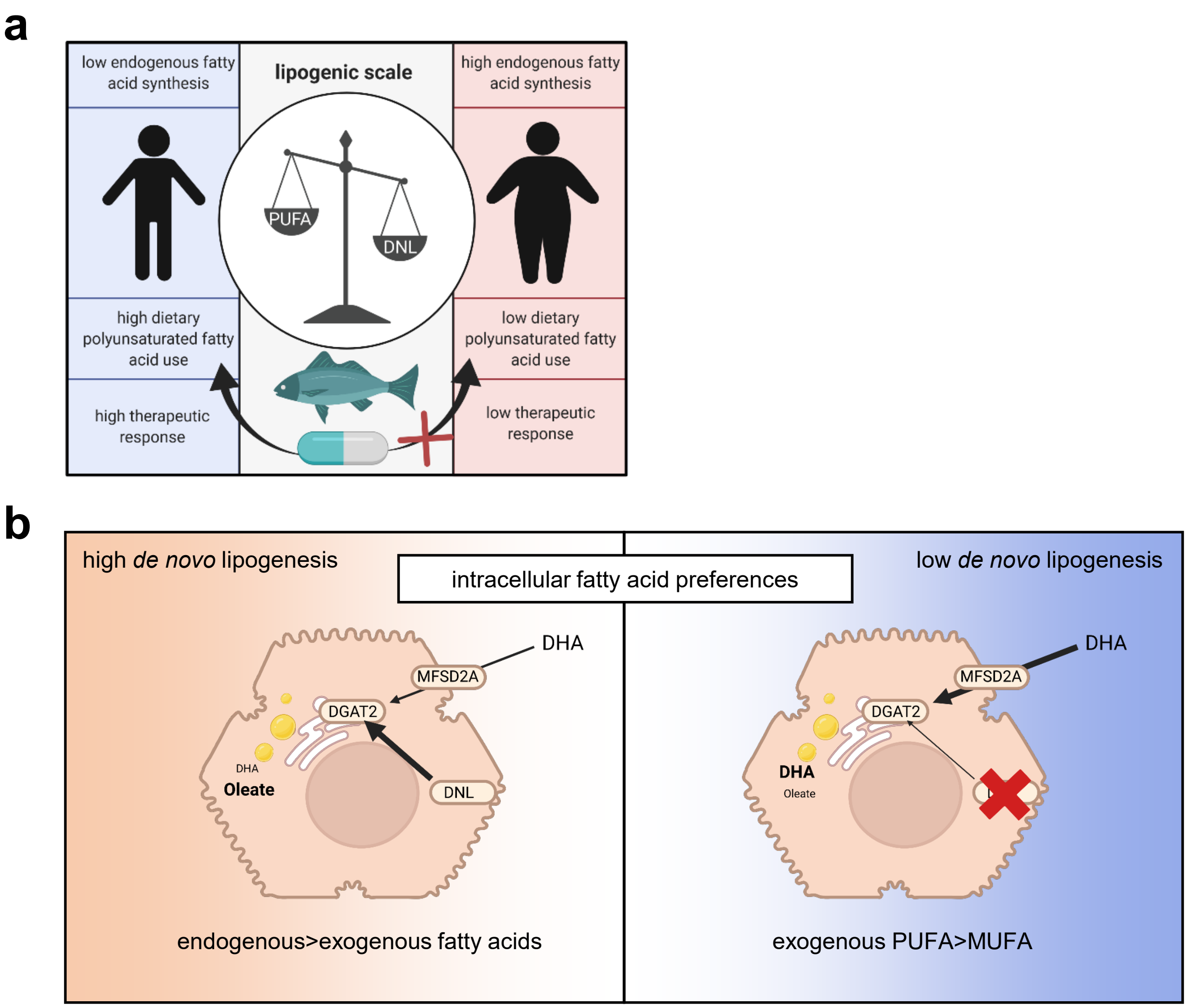

Fig.S16: Working model. (a) Model of PUFA use in lean respectively obese patients. Endogenous fatty acids derived from *de novo* lipogenesisceratedwith (DNL) suppress the incorporation of exogenous PUFA and might lead to reduced therapeutic responses. Figure was created with BioRender.com (b) High *de novo* lipogenesis supersedes the use of PUFA. When blocking lipogenesis, exogenous PUFAs are preferentially used for storage. Figure was created with BioRender.com

Supplemental References

1. Kircher, M., *et al.* A general framework for estimating the relative pathogenicity of human genetic variants. *Nat Genet* **46**, 310-315 (2014).
