# Supplementary material for: Fatty acid synthesis suppresses dietary polyunsaturated fatty acid use
Source: Nat Commun. 2024 Jan 2;15:45. doi: 10.1038/s41467-023-44364-y (PMC10762034; doi:10.1038/s41467-023-44364-y)
Supplement: Supplementary file 3 — Reporting Summary [file 41467_2023_44364_MOESM3_ESM.pdf]

nature portfolio

Corresponding author(s): Schlein, Christian

Last updated by author(s): Nov 17, 2023

Reporting Summary

Nature Portfolio wishes to improve the reproducibility of the work that we publish. This form provides structure for consistency and transparency in reporting. For further information on Nature Portfolio policies, see our Editorial Policies and the Editorial Policy Checklist.

Statistics

For all statistical analyses, confirm that the following items are present in the figure legend, table legend, main text, or Methods section.

n/a	Confirmed	
☐	☒	The exact sample size (*n*) for each experimental group/condition, given as a discrete number and unit of measurement
☐	☒	A statement on whether measurements were taken from distinct samples or whether the same sample was measured repeatedly
☐	☒	The statistical test(s) used AND whether they are one- or two-sided<br>*Only common tests should be described solely by name; describe more complex techniques in the Methods section.*
☒	☐	A description of all covariates tested
☐	☒	A description of any assumptions or corrections, such as tests of normality and adjustment for multiple comparisons
☐	☒	A full description of the statistical parameters including central tendency (e.g. means) or other basic estimates (e.g. regression coefficient) AND variation (e.g. standard deviation) or associated estimates of uncertainty (e.g. confidence intervals)
☐	☒	For null hypothesis testing, the test statistic (e.g. *F*, *t*, *r*) with confidence intervals, effect sizes, degrees of freedom and *P* value noted<br>*Give P values as exact values whenever suitable.*
☒	☐	For Bayesian analysis, information on the choice of priors and Markov chain Monte Carlo settings
☒	☐	For hierarchical and complex designs, identification of the appropriate level for tests and full reporting of outcomes
☒	☐	Estimates of effect sizes (e.g. Cohen's *d*, Pearson's *r*), indicating how they were calculated

Our web collection on statistics for biologists contains articles on many of the points above.

Software and code

Policy information about availability of computer code

Data collection	Microsoft Excel 365
Data analysis	Lipidomics Workflow Manager software version 1.0.5.0; SCIEX, Graphpad Prism 9, Perseus Version 1.5.8.5., Proteome Discoverer 3.0.0.757

For manuscripts utilizing custom algorithms or software that are central to the research but not yet described in published literature, software must be made available to editors and reviewers. We strongly encourage code deposition in a community repository (e.g. GitHub). See the Nature Portfolio guidelines for submitting code & software for further information.

Data

Policy information about availability of data

All manuscripts must include a data availability statement. This statement should provide the following information, where applicable:

- Accession codes, unique identifiers, or web links for publicly available datasets
- A description of any restrictions on data availability
- For clinical datasets or third party data, please ensure that the statement adheres to our policy

The mass spectrometry proteomics data have been deposited to the ProteomeXchange Consortium via the PRIDE partner repository with the dataset identifier PXD045908. The data of clinical trial NCT03938246 that support the findings of this study are available from Sagimet but restrictions apply to the availability of these data, which were used under license for the current study, and so are not publicly available. Data are however available from the authors upon reasonable request and with permission of Sagimet. All other relevant data of this study are available within the article and its supplementary information file. Source data for Figures 1-4 are provided with this paper.

Research involving human participants, their data, or biological material

Policy information about studies with human participants or human data. See also policy information about sex, gender (identity/presentation), and sexual orientation and race, ethnicity and racism.

Reporting on sex and gender	The participants were recruited independent of sex or gender. The index patient and the corresponding controls were male. The clinical trial NCT03938246 recruited male and female individuals, details can be found at PMID 34310978.
Reporting on race, ethnicity, or other socially relevant groupings	The index patient and his parents are from Kongo and Angola. Details to self reported ethnicity of clinical trial participants can be found at PMID 34310978.
Population characteristics	The index patient presented at the age of 5 years, the father was 54. FASCINATE-1 is a randomized 12-week placebo-controlled study of the FASN inhibitor TVB-2640 in NASH at 10 US sites. The study population included male and female subjects aged 18 years and older. Median age of the placebo group was 52 years and of the TVB-2640 group was 55 years. Median BMI of the placebo group was 31.2 and of the TVB-2640 group was 32.8. Within the placebo group, 45.2% of the participants were male and 62.9% of the TVB-2640 group were male participants. See also PMID 34310978.
Recruitment	The index patient, the parents and all controls were recruited at the University Medical Center Hamburg-Eppendorf. No biases were intended or observed in respect of volounteering for the study. For the FASCINATE-1 study, adults with ≥8% liver fat, assessed by magnetic resonance imaging proton density fat fraction, and evidence of liver fibrosis by magnetic resonance elastography ≥2.5 kPa or liver biopsy were eligible. See also PMID 34310978.
Ethics oversight	All research presented in this study complies with the relevant ethical regulations. In particular, analysis involving material from study participants were carried out with written informed consent, and in case of children with written, informed consent of their legally authorized representatives and were conducted in accordance with the Declaration of Helsinki, and approved by the Ärtzekammer Hamburg, Germany. Additionally, data was derived from the clinical study NCT0393824610 that was conducted in accordance with ethical principles of the Declaration of Helsinki and consistent with the International Conference on Harmonization, Good Clinical Practice, and applicable regulatory requirements.

Note that full information on the approval of the study protocol must also be provided in the manuscript.

Field-specific reporting

Please select the one below that is the best fit for your research. If you are not sure, read the appropriate sections before making your selection.

☒ Life sciences ☐ Behavioural & social sciences ☐ Ecological, evolutionary & environmental sciences

For a reference copy of the document with all sections, see nature.com/documents/nr-reporting-summary-flat.pdf

Life sciences study design

All studies must disclose on these points even when the disclosure is negative.

Sample size	No sample size calculation was performd. For the index patient and his father n=1 (as no other patient with this specific de novo variant was available); Based on experiences of previous studies, mouse experiments were routinely performed with n=5 or more mice were used per group as indicated in the methods and/or figure legends. Similarly, based on previous studies, cell culture experiments were performed in at least three biological replicate experiments. Exact number of all sample sizes are given in the figure legends.
Data exclusions	Pre-established exclusion criteria were performed via Grubbs's test for outliers.
Replication	In accordance to the 3R priciples, no animal experiments were technically repeated. All cell culture experiments were independently performed at least three or more times with the same outcome.
Randomization	All mice of the animal studies were age and sex matched and afterwards randomly assigned to the groups. Experimental groups in cell culture experiments were allocated randomly. For the FASCINATE-1 study, subjects were randomly assigned to TVB-2640 or placebo group (see also

	PMID 34310978).
Blinding	Cell culture and animal studies were perforemd without blinding in order to simplify experiments for the experimentators for practical reasons. For the FASCINATE-1 study, personnel responsible for reading and interpreting MRI-PDFF images were blinded to the subjects' treatment assignment group (see also PMID 34310978).

Reporting for specific materials, systems and methods

We require information from authors about some types of materials, experimental systems and methods used in many studies. Here, indicate whether each material, system or method listed is relevant to your study. If you are not sure if a list item applies to your research, read the appropriate section before selecting a response.

Materials & experimental systems

n/a	Involved in the study
☐	☒ Antibodies
☐	☒ Eukaryotic cell lines
☒	☐ Palaeontology and archaeology
☐	☒ Animals and other organisms
☐	☒ Clinical data
☒	☐ Dual use research of concern
☒	☐ Plants

Methods

n/a	Involved in the study
☒	☐ ChIP-seq
☒	☐ Flow cytometry
☒	☐ MRI-based neuroimaging

Antibodies

Antibodies used	Acetyl-Lys (Cell Signaling; 9441S; 1:500 dilution), AKT (Cell Signaling; 9272S; 1:1000 dilution), Calreticulin: (Cell Signaling: #4850; 1:1000.dilution), CHOP (Cell Signaling; 2895; 1:1000 dilution), COXIV (Cell Signaling; #4850; 1:1000 dilution), E-Cadherin (Cell Signaling; #3195; 1:1000 dilution), FASN (BD Biosciences; 610962; 1:1000 dilution), GAPDH (Santa Cruz; Sc32233; 1:15000 dilution), γ-tubulin (abcam; Ab179503; 1:2000 dilution), HA (Roche; 12013819001; 1:10000 dilution), Histon-H3 (abcam; Ab4729; 1:1000 dilution), P-Ser (Sigma-Aldrich; AB1603; 1:1000 dilution), RCAS (Cell Signaling; #12290; 1:1000 dilution), LDHA (Cell Signaling; 2012; 1:1000 dilution), Malonyl-Lys (Cell Signaling; #14942; 1:1000 dilution), c-MYC (Sigma Aldrich; M5546; 1:100 dilution), SREBP (Invitrogen; MA5-11685; 1:1000 dilution), Ubiquitin (Merck; MAB1510-I; clone Ubi-1; 1:1000 dilution)<br>donkey anti-rabbit IgG Horseradish Peroxidase secondary antibody (GE Healthcare; no. NA934V; 1:7,500 dilution), sheep anti-mouse IgG Horseradish Peroxidase secondary antibody (GE Healthcare; no. NA931V; 1:7,500 dilution).
Validation	Anti-Acetyl-Lysine, Anti-Malonyl-Lysine, Anti-Calreticulin, Anti-CHOP, Anti-COXIV, Anti-E-Cadherin, Anti-RCAS, and Anti-AKT antibodies were validated by Cell Signaling with positive and negative controls (such as (non-)acetylated BSA (#9441), cross reactivity with other lysine modifications (#14942) and #9272 siRNA approaches). Anti-HA and Anti-FASN was additionally validated by our overexpression approaches. Anti-Ubiquitin was additionally validated by our proteasome inhibition apporaches. All other antibodies were validated using positive controls by their respective supplier.

Eukaryotic cell lines

Policy information about cell lines and Sex and Gender in Research

Cell line source(s)	Fibroblasts derived from male clinical patients; HEK293T cells purchased from ATCC (2017). WT and DGAT2-KO MEFs were a gift of the Walther/Farese lab (Harvard Medical School, Boston, USA), HuH7 cells were purchased from CLS (2022).
Authentication	None of the cells were additionally authenticated after purchase. MEFS were genotyped for the lack of DGAT2 by gene expression analysis.
Mycoplasma contamination	All cell lines were tested negative for mycoplasma contamination.
Commonly misidentified lines<br>(See ICLAC register)	none

Animals and other research organisms

Policy information about studies involving animals; ARRIVE guidelines recommended for reporting animal research, and Sex and Gender in Research

Laboratory animals	ChREBP-KO (Mlxipl-/-) or wild type littermates between 8 and 20 weeks on C57Bl6/J background, wild type C57BL6/J animals or FASNR1812W animals were kept in single cages in climate chambers (Memmert) with ad libitum access to regular chow diet (Altromin, 1329P) and water. Mice were housed at room temperature (22°C) with a day and night cycle of 12 h each and a humidity of 50 %.
Wild animals	The study did not involve wild animals

Reporting on sex	Age and sex matched male and female mice were used.
Field-collected samples	The study did not involve field-collected samples
Ethics oversight	All animal studies were performed with permission of the Animal Welfare Officers at University Medical Center Hamburg-Eppendorf. Studies at Washington University were approved by the institutional Animal Studies Committee.

Note that full information on the approval of the study protocol must also be provided in the manuscript.

Clinical data

Policy information about clinical studies

All manuscripts should comply with the ICMJEguidelines for publication of clinical research and a completedCONSORT checklist must be included with all submissions.

Clinical trial registration	ClinicalTrials.gov, Number NCT03938246
Study protocol	Study protocol is available upon reasonable request.
Data collection	Randomized, placebo-controlled, single-blind study April 2019-August 2020. at:<br><br>Chula Vista, California, United States, 91911 ProSciento;<br>Montclair, California, United States, 91763, Catalina Research Institute<br>Sacramento, California, United States, 95821,Clinical Trials Research<br>San Diego, California, United States, 92037,University of California San Diego (UCSD)<br>Miami Lakes, Florida, United States, 33014 Panax<br>Morehead City, North Carolina, United States, 28557Lucas Research<br>Austin, Texas, United States, 78749,Texas Diabetes and Endocrinology - Austin<br>Cedar Park, Texas, United States, 78613, Texas Digestive Disease Consultants - Cedar Park<br>Dallas, Texas, United States, 75246,Texas Digestive Disease Consultants - Dallas<br>Fort Worth, Texas, United States, 76104,Texas Digestive Disease Consultant - Ft Worth<br>San Antonio, Texas, United States, 78229, Pinnacle Clinical Research - San Antonio<br>Webster, Texas, United States, 77598,Texas Digestive Disease Consultants - Webster
Outcomes	Primary:<br>• To determine the effect of once daily (QD) TVB-2640 for 12 weeks versus placebo on the change in hepatic fat fraction by proton density fat fraction magnetic resonance imaging (MRI-PDFF) from baseline in subjects with non-alcoholic steatohepatitis (NASH).<br>• To determine the safety of QD TVB-2640 versus placebo in subjects with NASH, including the effects on alanine aminotransferase (ALT) and aspartate aminotransferase (AST).<br>Secondary:<br>• To determine the effect of once-daily (QD) TVB-2640 for 12 weeks versus placebo in subjects with NASH on:<br>- Lipid and lipoprotein parameters, including low-density lipoprotein cholesterol (LDL-C), non-LDL-C, high-density lipoprotein cholesterol (HDL-C), non-HDL-C, total cholesterol, triglycerides, apolipoprotein B (ApoB), and lipoprotein(a) (Lp[a]) particles.<br>- NASH and fibrosis biomarkers including cytokeratin-18 (CK-18), fibrosis-4 (FIB-4), and enhanced liver function (ELF) or FIBROSpect 2 or ProC3 test.<br>- Eicosanoid panel.<br>Exploratory:<br>• To determine the effect of QD TVB-2640 for 12 weeks versus placebo in subjects NASH on clinical measures, including:<br>- Liver fibrosis, as determined by vibration-controlled transient elastography (VCTE).<br>- Metabolic and inflammatory parameters, including fasting glucose, insulin, homeostatic model assessment insulin resistance (HOMA2-IR), glycated hemoglobin (HbA1c), non-esterified fatty acid (NEFA), adipose tissue insulin resistance (adipo-IR), adiponectin, resistin, Interleukin 6 (IL-6), and gamma-glutamyl transpeptidase (GGT).<br>- Anthropometric parameters, including weight, waist and hip circumference, waist-hip ratio, and blood pressure.<br>- Lipodomic analyses for de novo lipogenesis (DNL). [>>> note: focus of the manuscript]<br>• Explore the relationship of plasma drug exposure to changes in efficacy and safety biomarkers.<br>• Explore possible relationships between genomic markers of NASH and responses to treatment.
